# Supplementary material for: Observation of aligned dipoles and angular chromism of exciplexes in organic molecular heterostructures
Source: Nat Commun. 2023 Nov 8;14:7190. doi: 10.1038/s41467-023-42976-y (PMC10632441; doi:10.1038/s41467-023-42976-y)
Supplement: Supplementary file 1 — Supplementary Information [file 41467_2023_42976_MOESM1_ESM.pdf]

Supplementary Information

Observation of aligned dipoles and angular chromism of exciplexes in organic molecular heterostructures

Sang-hun Lee[1], Taek Joon Kim[1], Eunji Lee[2], Dayeong Kwon[1], Jeongyong Kim[2,*], Jinsoo Joo[1,*]

Affiliations

[1] Department of Physics, Korea University, Seoul 02841, Republic of Korea

[2] Department of Energy Science, Sungkyunkwan University, Suwon 16419, Republic of Korea

Corresponding authors

Jinsoo Joo (j.joo@korea.ac.kr) and Jeongyong Kim (j.kim@skku.edu)

Supplementary Note 1: LCM PL and tr-PL spectra of m-MTDATA layer

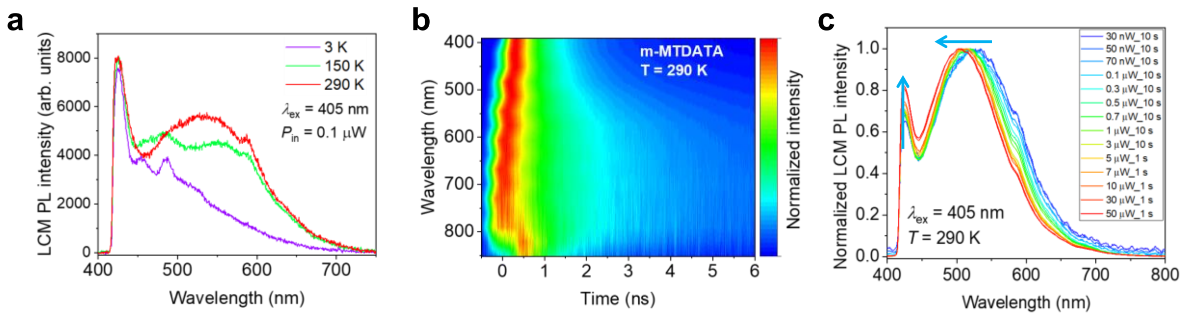

Supplementary Fig. 1 | LCM PL spectra of m-MTDATA. a LCM PL spectra of m-MTDATA layer at various temperatures (T = 3, 150, and 290 K) with λ_{ex} = 405 nm and P_{in} = 0.1 μW. **b** 16-channel tr-PL spectra of m-MTDATA layer at 290 K with λ_{ex} = 375 nm. **c** Normalized LCM PL spectra of m-MTDATA layer with various excitation power (P_{in}) from 30 nW to 50 μW at 290 K (λ_{ex} = 405 nm).

Supplementary Fig. 1a shows the LCM PL spectra of the m-MTDATA layer at 3 K (purple), 150 K (green), and 290 K (red). As the temperature increases, the PL emission (λ_{em} ~ 530 nm) from the excimers of m-MTDATA ($XM_{m\text{-}MT}$) relatively increases. Supplementary Fig. 1b shows 16-channel tr-PL decay curves. The decay time of $XM_{m\text{-}MT}$ (λ_{em} = 528 nm at 290 K) was longer than that of $XF_{m\text{-}MT}$ (λ_{em} = 425 nm at 290 K) in the m-MTDATA layer. Supplementary Fig. 1c shows the normalized LCM PL spectra of the m-MTDATA layer at various excitation powers (P_{in}) at 290 K. The $XM_{m\text{-}MT}$ peaks (λ_{em} = 528 nm at 290 K) were blue-shifted with increasing P_{in}, whereas the $XF_{m\text{-}MT}$ peak position (λ_{em} = 427 nm) did not shift.

Supplementary Note 2: LCM PL spectra of m-MTDATA and T2T layers

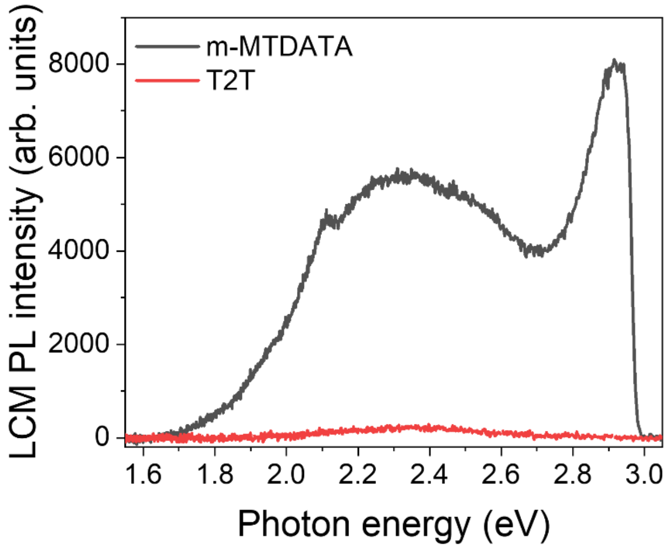

Supplementary Fig. 2 | LCM PL spectra of m-MTDATA and T2T. Comparison of LCM PL spectra of m-MTDATA (black curve) and T2T (red curve) layers on Si/SiO_2 at 290 K (λ_{ex} = 405 nm, P_{in} = 0.1 μW).

Supplementary Fig. 2 shows the PL spectra of the m-MTDATA and T2T layers (λ_{ex} = 405 nm, P_{in} = 0.1 μW). Because the T2T molecules were barely absorbed the excitation laser (λ_{ex} = 405 nm), the LCM PL intensity of T2T was considerably weaker than that of m-MTDATA.

Supplementary Note 3: Optical absorption and UPS spectra

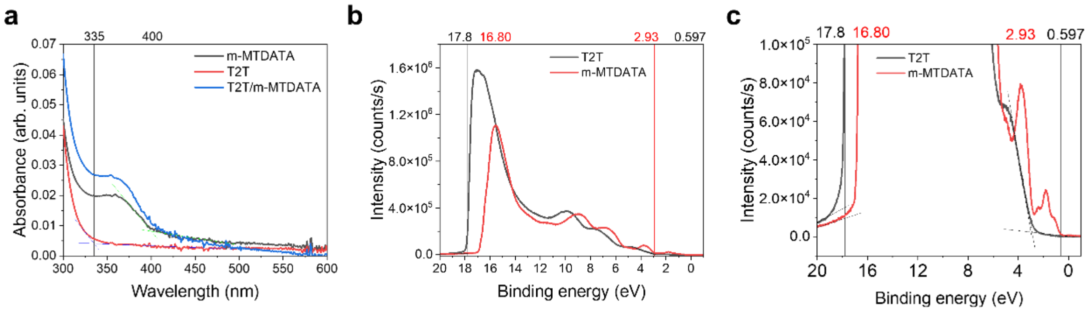

Supplementary Fig. 3 | Optical absorption and UPS spectra. a Optical absorption spectra of m-MTDATA (black curve) layer, T2T (red curve) layer, and T2T/m-MTDATA (blue curve) bilayer. **b** UPS results of m-MTDATA (black curve) and T2T (red curve). **c** Magnification of Supplementary Fig. 3b.

Optical absorption and UPS spectra were recorded to estimate the HOMO and LUMO levels of m-MTDATA, T2T, and their bilayers (BL). The HOMO levels were calculated as per $E_{\mathrm{HOMO}} = h\nu - (E_{\mathrm{cut\text{-}off}} - E_{\mathrm{rel}})$, where $h\nu$ and E_{HOMO} are the energy of the excitation source (21.22 eV) and the energy offset from the vacuum level to the HOMO level, respectively. Further, $E_{\mathrm{cut\text{-}off}}$ and E_{rel} represent the secondary electron cut-off and relative HOMO energies, respectively. Therefore, the subtraction term, $E_{\mathrm{cut\text{-}off}} - E_{\mathrm{rel}}$, implies the difference of edges of a UPS spectrum. The LUMO levels were estimated using $E_{\mathrm{LUMO}} = E_{\mathrm{HOMO}} + E_{\mathrm{g}}$, where E_{g} is the energy gap. For the m-MTDATA (T2T) layer, $E_{\mathrm{cut\text{-}off}}$ and E_{rel} were estimated to be 16.80 eV (17.80 eV) and 0.597 eV (2.93 eV), respectively, as shown in Supplementary Fig. 3b and c. The HOMO levels (E_{HOMO}) of m-MTDATA and T2T were estimated to be 5.00 and 6.33 eV, respectively. The LUMO levels (E_{LUMO}) were obtained by the summation of optical band gap (absorption spectrum edge): 3.10 and 3.70 eV for m-MTDATA and T2T, respectively. The E_{LUMO} of m-MTDATA and T2T were estimated as 1.90 and 2.63 eV, respectively. The energy-band alignment of the E_{HOMO} and E_{LUMO} for the m-MTDATA/T2T bilayer is shown in Fig. 1d.

Supplementary Note 4: BFP mapping of m-MTDATA/T2T bilayer (BL)

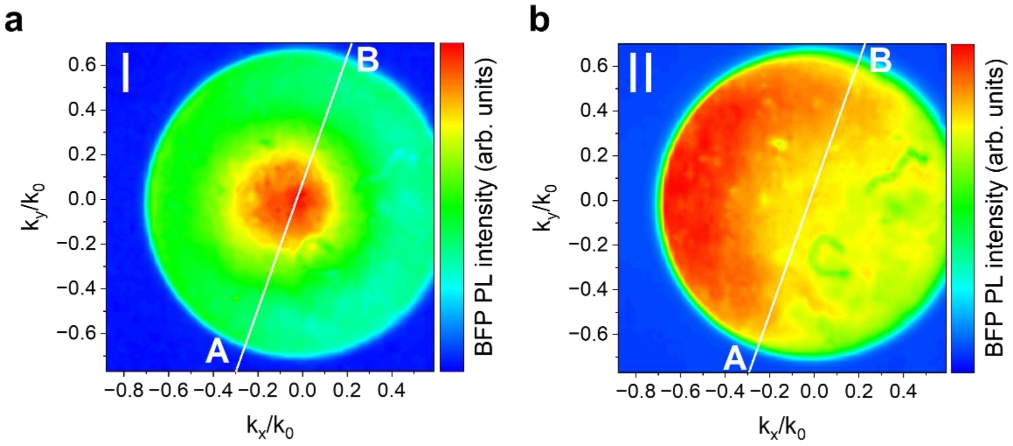

Supplementary Fig. 4 | BFP mapping of m-MTDATA/T2T BL. BFP PL mapping images of **a** $XF_{m\text{-}MT}$ (region I in Fig. 2d) and **b** $XP_{m\text{-}MT/T2T}$ (including XM; region II in Fig. 2d). Regions I and II correspond to λ = 420–430 and 520–650 nm, respectively, in Fig. 2d. The slight gradient along the diagonal of the BFP image attributed to the imperfect alignment of the scanning plane during BFP mapping.

Supplementary Note 5: BFP PL of m-MTDATA:T2T co-deposition layer (CDL)

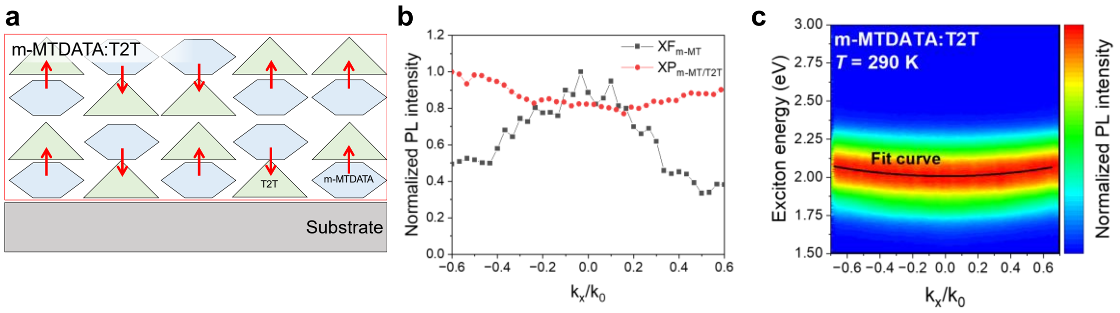

Supplementary Fig. 5 | BFP PL of m-MTDATA:T2T CDL. a Schematic illustration of random molecular distribution for m-MTDATA:T2T CDL. The red arrows indicate the schematic direction of $XP_{m\text{-}MT:T2T}$ in the CDL. **b** Normalized PL intensity of $XF_{m\text{-}MT}$ (black) and $XP_{m\text{-}MT/T2T}$ (red) as a function of k_x/k_0. **c** Normalized momentum (k_x/k_0)-dependent BFP PL spectra (exciton energy) of m-MTDATA:T2T CDL with parabolic fitting curves at 290 K.

Supplementary Note 6: Deconvolution of LCM PL spectra

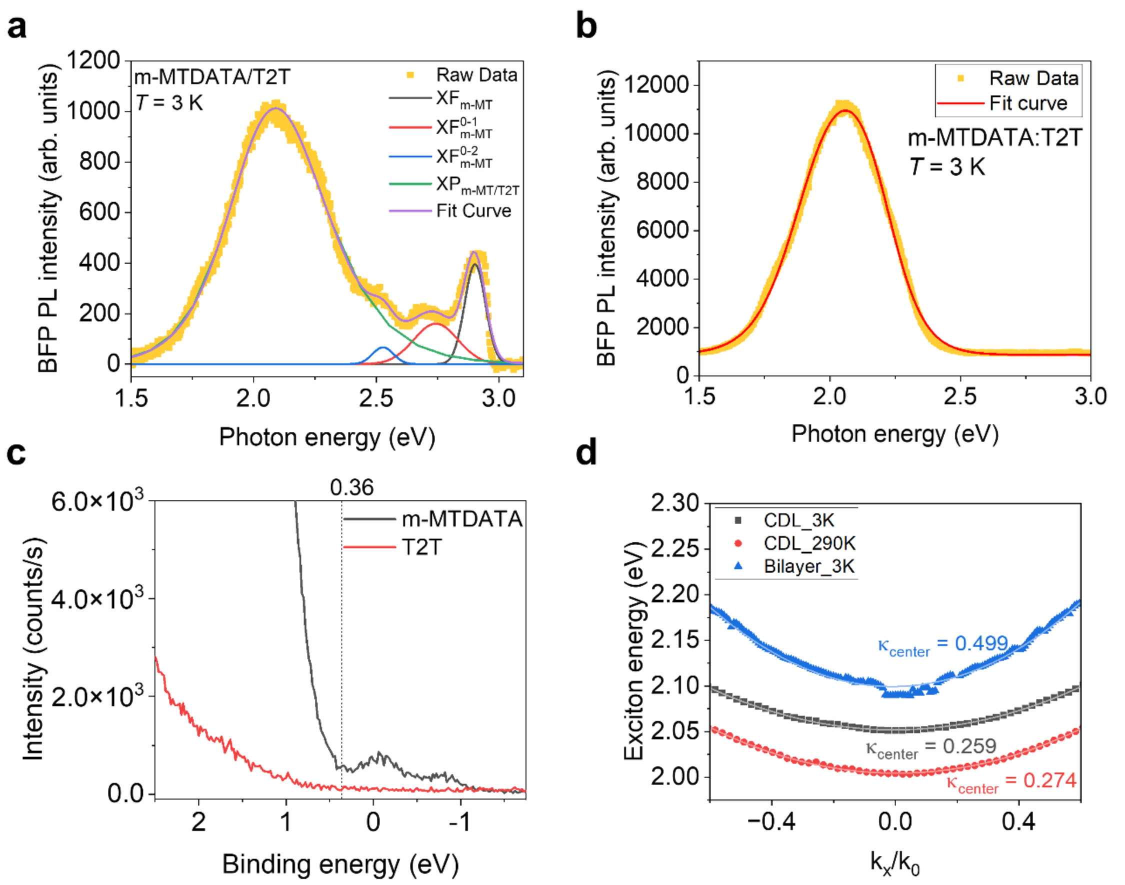

Supplementary Fig. 6 | Deconvolution of LCM PL spectra. BFP PL spectrum with deconvoluted curves for **a** m-MTDATA/T2T BL and **b** m-MTDATA:T2T CDL. **c** Magnification of UPS spectra of m-MTDATA (black curve) and T2T (red curve) layer. **d** Parabolic fitting result of BFP PL peak position of $XP_{m\text{-}MT:T2T}$ in the CDL at 3 K (black), CDL at 290 K (red), and $XP_{m\text{-}MT/T2T}$ in the BL at 3 K (blue).

The BFP PL spectrum of the m-MTDATA/T2T BL at 3 K (λ_{ex} = 405 nm) is deconvoluted, as shown in Supplementary Fig. 6a. The spectrum was deconvoluted via three Gaussian functions (for $XF_{m\text{-}MT}$, $XF^{0\text{-}1}_{m\text{-}MT}$, and $XF^{0\text{-}2}_{m\text{-}MT}$) and one asymmetric double sigmoidal function (Asym2SigFunc)[1,2]. In the deconvoluted curves in Supplementary Fig. 6a, the PL peaks at 2.90

eV (427.6 nm), 2.74 eV (452.6 nm), 2.53 eV (490.1 nm) and 2.09 eV (593.3 nm) correspond to the $XF_{m\text{-}MT}$, first vibrational mode of m-MTDATA ($XF^{0\text{-}1}{}_{m\text{-}MT}$), second vibrational mode of m-MTDATA ($XF^{0\text{-}2}{}_{m\text{-}MT}$), and $XP_{m\text{-}MT/T2T}$, respectively, at 3 K. The asymmetric PL spectrum of the long tail in the low-energy regime is explained by the valence band tail, that is, extra energy states near the valence band maximum owing to disorder. Supplementary Fig. 6b shows the BFP PL spectrum of m-MTDATA:T2T CDL (λ_{ex} = 405 nm) at 3 K. The spectrum was deconvoluted via one Asym2SigFunc (peak position = 2.05 eV). Supplementary Fig. 6c shows a magnified view of the UPS results for the m-MTDATA and T2T layers. Extra energy states owing to disorder were observed over the HOMO (black dotted line at 0.36 eV) of the m-MTDATA layer. As the temperature increased from 3 to 290 K, the curvature at the center (κ_{center}) increased from 0.259 to 0.274 because of the enhanced dispersion of $XP_{m\text{-}MT/T2T}$ in the CDL at higher temperatures. The κ_{center} of the m-MTDATA/T2T BL at 3 K was estimated as 0.499, as shown in Supplementary Fig. 6d.

Supplementary Note 7: Energy–momentum (*E*-*k*) dispersion of BL, CDL, drop-cast, reprecipitated films

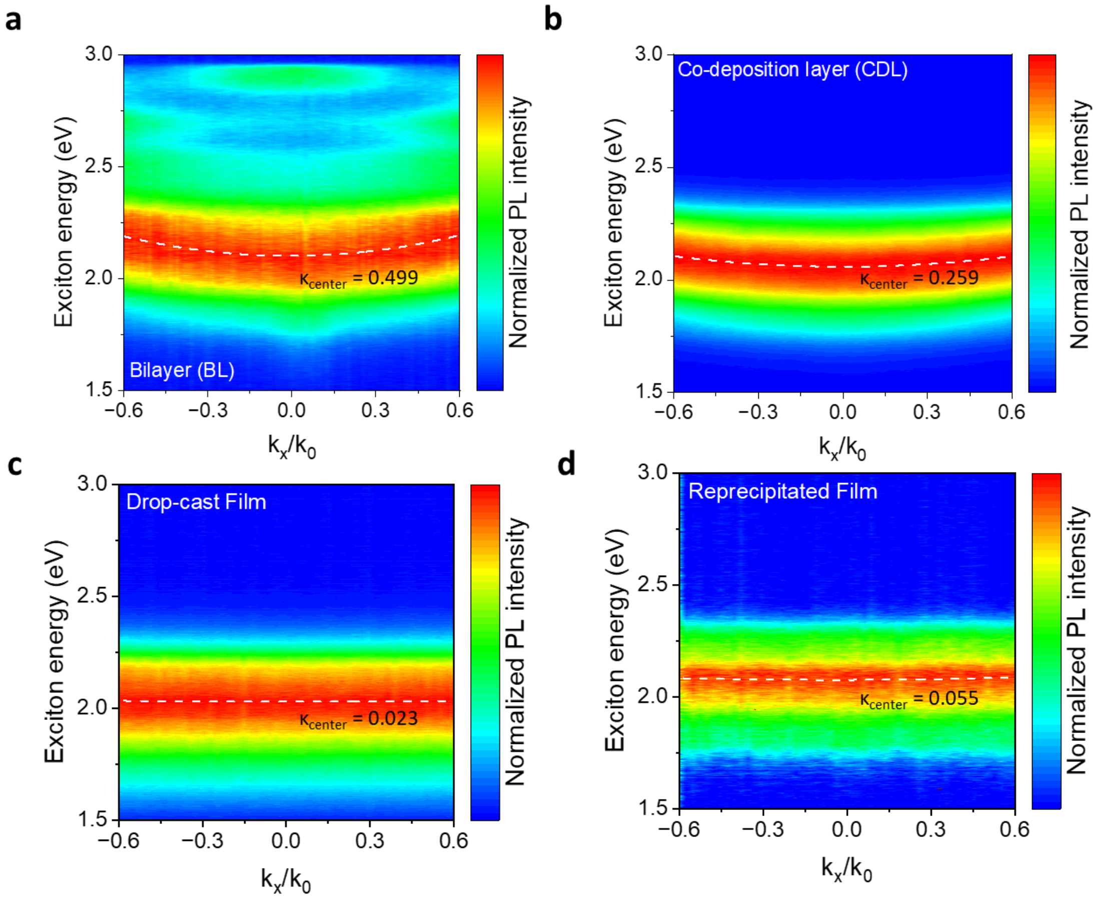

Supplementary Fig. 7 | Energy–momentum (*E*–*k*) dispersion. *E*-*k* dispersion of **a** BL, **b** CDL, **c** drop-cast film of m-MTDATA-T2T blended molecules, and **d** reprecipitated film of m-MTDATA-T2T blended molecules. White dashed lines are parabolic fitting curves.

To investigate the relationship between the XP energy-momentum (*E*-*k*) dispersion and the degree of dipole alignment of the XPs, we prepared a more disordered donor and acceptor blended film and performed the BFP measurement. Supplementary Fig. 7c shows *E* vs. *k* of the drop-cast film of the blended m-MTDATA-T2T molecules. Supplementary Fig. 7d shows *E*

vs. *k* of the reprecipitated film of the blended m-MTDATA-T2T molecules. The drop-cast and the reprecipitated films could be treated as heavily disordered molecular systems, which were made by drop-casting on a hotplate at 120 °C and injecting in deionized water (with vigorous stirring) of the tetrahydrofuran (THF) solution with m-MTDATA and T2T mixture (weight ratio of m-MTDATA and T2T is 1:1), respectively, on Si/SiO_2 substrates. Although the photon energy of XP is identical to that of our BL, the *E* vs. *k* relations of the XP peak from the BFP PL spectra of the drop-cast and reprecipitated films show nondispersive flat characteristics because of the severely random distribution of XP dipole orientations. This control experiment results confirm that the observed *E*-*k* dispersion characteristics of the BL originate from the broad overlap of the wave functions (delocalization) of the XPs in the BL. Their dispersive characters are quantitatively expressed using the estimated curvature $\kappa_{\text{center}} \equiv d^2E/dk^2$ in the Supplementary Table 1. The values of κ_{center} of disordered m-MTDATA:T2T blended films of drop-cast or reprecipitated films are negligible compared to $\kappa_{\text{center}}$of BL and CDL, showing no dispersive characteristics of *E* vs. *k* relation.

Supplementary Table 1 | $\boldsymbol{\kappa}_{\textbf{center}}$ values of XP in various HS samples.

Sample	BL	CDL	Drop-cast film	Reprecipitated film
κ_{center}	0.499	0.259	0.023	0.055

Supplementary Note 8: BFP PL images of XPs in BL, CDL, drop-cast film, and reprecipitated film

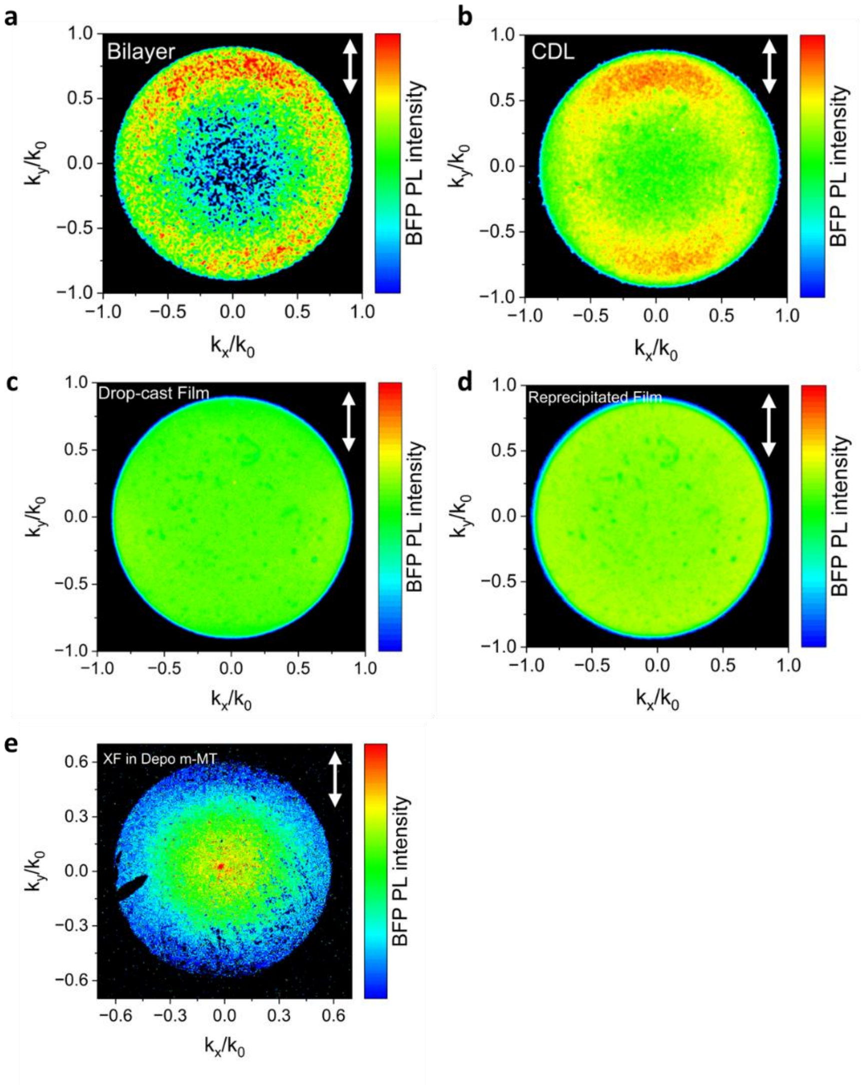

Supplementary Fig. 8 | Linearly polarized BFP PL images. BFP PL images of XPs in **a** BL, **b** CDL, **c** drop-cast film, and **d** reprecipitated film, and of **e** XF in deposited m-MTDATA. White arrows indicate direction of linear polarization. XP emissions were filtered using 488-nm long-pass filter, and XF emission was filtered using 409-nm long-pass and 450-nm short-pass filters. It is noted that the measurement system of these BFP (for Supplementary Fig. 8**a-d**) is different from that for Fig. 2 and Supplementary providing slightly larger range of k_x.

To provide the even more compelling experimental evidence, we performed the additional BFP imaging experiments by using a linear polarizer in front of the spectrometer and resolve the p- and s-polarized emissions on the BFP. The exciton dipole moments vertical to the substrate should predominantly emit p-polarized (p-pol) light[3]. A linear polarizer was placed in front of the detector to observe the XP characteristics in the BL and CDL samples. Long-pass and short-pass optical filters were used to observe the target emissions (XP or XF), and the intensity profile at the BFP was observed using a CCD camera. In addition, we performed additional BFP experiments using differently prepared samples of the drop-cast film and the film prepared by the reprecipitation method (reprecipitated film) of blended m-MTDATA-T2T molecules, which were more disordered than the slowly deposited m-MTDATA/T2T BL. The BFP images of the pristine m-MTDATA was measured for the comparison purpose. As shown in Supplementary Fig. 8a and b., BL and CDL samples showed p-polarization-dominant emission, indicating the vertical direction (out-of-plane) of the dipole moments. In contradict, the drop-cast m-MTDATA/T2T film and the reprecipitated film that are more disordered samples showed no polarized emission pattern of XPs, as shown in Supplementary Fig. 8c and d because of the random orientation of the XP dipoles.

Through a series of additional experiments with the BL, CDL, drop-cast film, and reprecipitated film resolving s- and p-polarization, we confirmed that the characteristic concavity of the cross-sectional profile across the BFP occurs only for the XPs of the BL or CDL and doesn’t occur for disordered sample systems (Supplementary Fig. 7). Note that the drop-cast film produces XPs at the same wavelength as the BL or CDL. Therefore, the systematic increase in the concavity of the BFP profile and angular chromism between the BL (or CDL) and drop-cast (or reprecipitated) films provides the strong evidence of the aligned dipoles of XP in the BL and CDL.

Supplementary Note 9: Refraction indices and cross-sectional SEM images

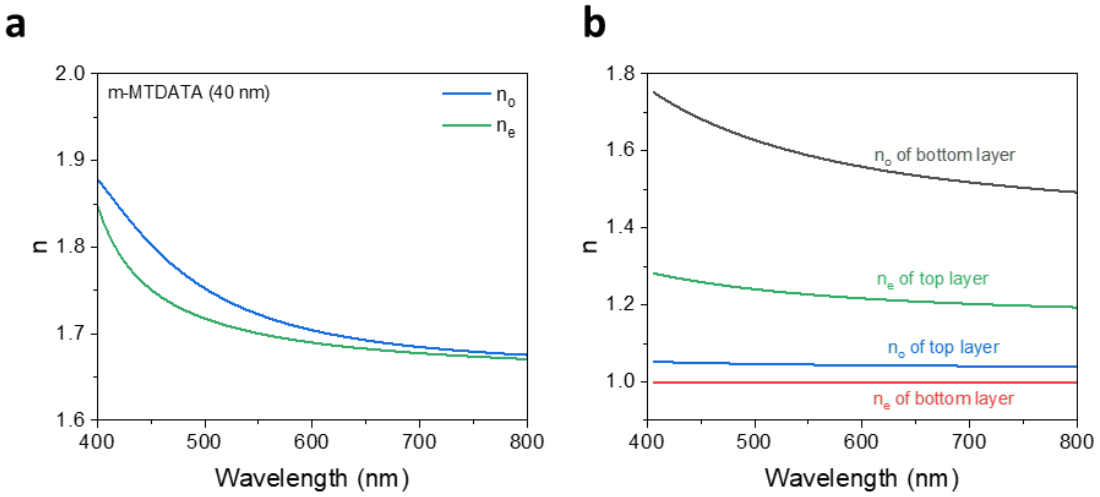

-

Supplementary Fig. 9 | Variable-angle spectroscopic ellipsometry. Calculated refraction indices of **a** m-MTDATA layer and **b** T2T on m-MTDATA layer as functions of wavelength obtained from spectroscopic ellipsometry measurements.

The ordinary (n_o) and extraordinary (n_e) refractive indices of m-MTDATA, T2T layers, and their BL as a function of wavelength were calculated using DeltaPsi2 software from the measured data of variable-angle spectroscopic ellipsometry (VASE) using an ellipsometer (UVISEL Plus, HORIBA), as shown in Supplementary Fig. 9. The n_o and n_e values of the m-MTDATA layer were calculated to be 1.72 and 1.70 (negative birefringence) at λ = 550 nm, which agrees with previous results[4]. These results indicated that the m-MTDATA molecules had a stacked in-plane columnar structure, agreeing with the previous result[5]. The calculation of n_o and n_e values of the T2T layer and m-MTDATA/T2T BL using OMBD should be considered more complicated because of the nanorod structure of the T2T molecules and their random deposition, as shown in Fig. 3a. Because the deposited configuration of the T2T molecules is non-uniform, the model of the exponential gradient layer with anisotropy and vacuum defects (voids) was used, which comprises the bottom and top layers with ordinary

and extraordinary terms, respectively. The calculation result of the T2T bottom layer of the m-MTDATA/T2T BL gave the n_o and n_e values as 1.59 and 1.0 (negative birefringence) at λ = 550 nm, respectively. The calculation result of the T2T top layer of the BL gave the n_o and n_e values as 1.04 and 1.23, respectively, (positive birefringence). The relatively lower values of n_o and n_e for the BL compared to a single layer of previous results[4] might be due to the random orientation of the T2T nanorods and the large air space between them.

The cross-sectional SEM and TEM images can support the analysis of ellipsometer calculations. The cross-sectional SEM images of the m-MTDATA, T2T, and m-MTDATA/T2T BL are shown in Supplementary Fig. 10. The m-MTDATA/T2T BLs were different batch samples with greater thicknesses. The cross-sectional SEM image confirmed the in-plane columnar stacking for the m-MTDATA layer (Supplementary Fig. 10a), whereas a random distribution of T2T molecules was observed (Supplementary Fig. 10b). Notably, the T2T molecules were stacked in-plane on the surface of m-MTDATA (i.e., at the interface), and then stacked with randomly standing nanorods on the outer surface, as shown in Supplementary Fig. 10c. The cross-sectional SEM images clearly demonstrate distinctive molecular stacking in the m-MTDATA/T2T BL. Interestingly, the in-plane interface between the m-MTDATA and T2T layers was clearly observed (Supplementary Fig. 11a and b), whereas there was not clear interface for the CDL as shown in Supplementary Fig. 11c and d.

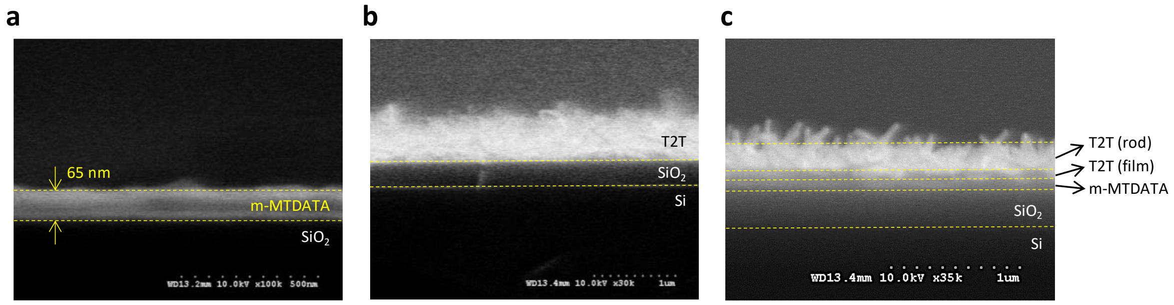

Supplementary Fig. 10 | Cross-sectional SEM images. **a** m-MTDATA, **b** T2T and **c** m-MTDATA/T2T BL.

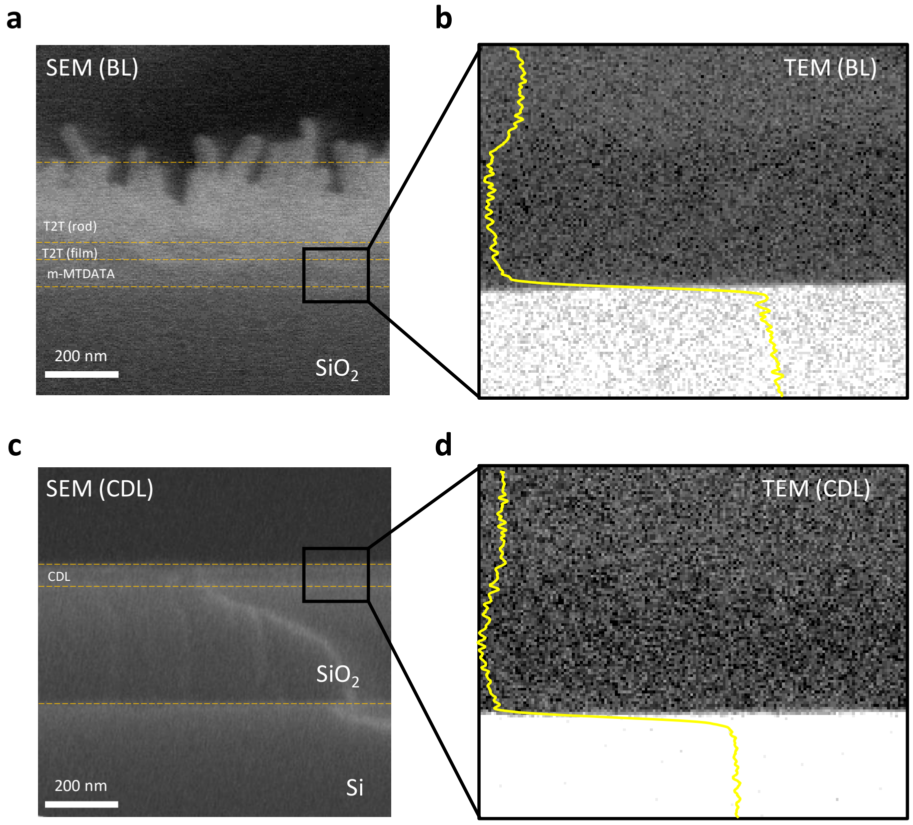

Supplementary Fig. 11 | Cross-sectional SEM and TEM images. Magnification of cross-sectional SEM and TEM images of (**a** and **b**) BL and (**c** and **d**) CDL of m-MTDATA and T2T, respectively. Yellow curves of corresponding TEM images represent the electron scattering intensity profiles.

In sum, for m-MTDATA/T2T BL, the results of ellipsometry experiments combined with the cross-sectional SEM and TEM images suggest that m-MTDATA and T2T molecules were in-plane stacking at the interface, above which the T2T molecules were randomly stacked out-of-plane at the top as the form of nanorods, as shown in Fig. 3 and Supplementary Fig. 10 and 11.

Supplementary Note 10: PL peak positions of m-MTDATA/T2T bilayer

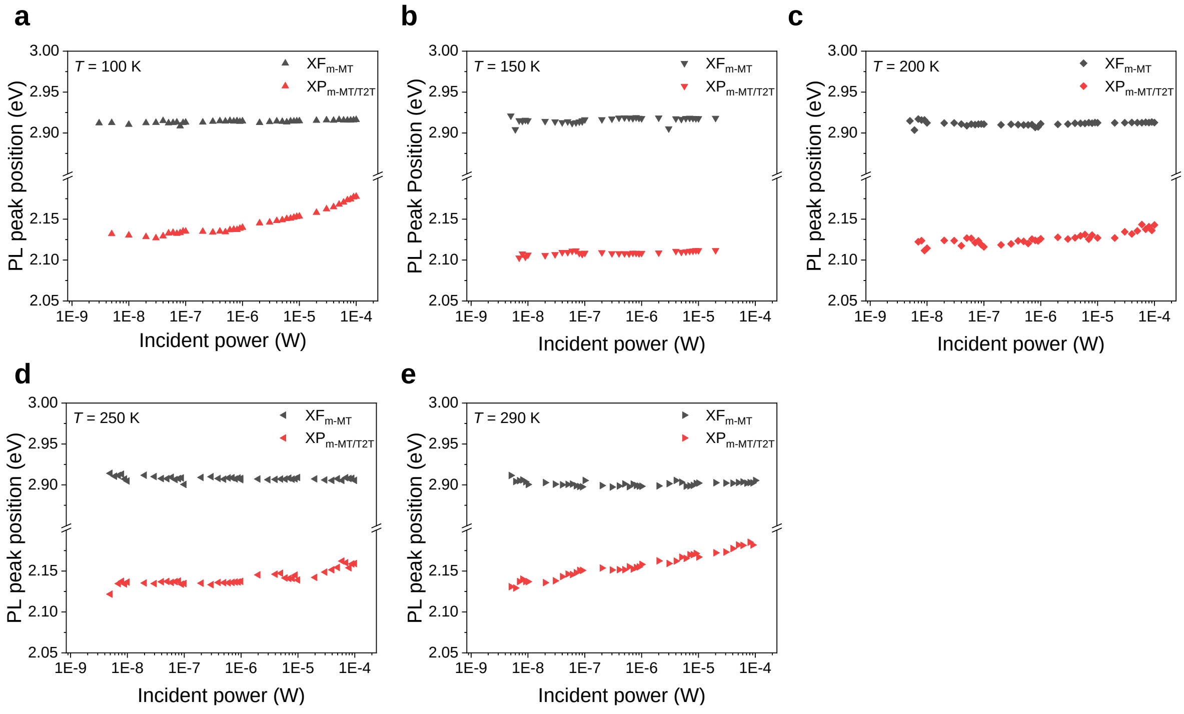

Supplementary Fig. 12 | PL peak positions of m-MTDATA/T2T bilayer. Incident excitation power (P_{in}) dependence of PL peak position of $XF_{m\text{-}MT}$ and $XP_{m\text{-}MT/T2T}$ from fitting results of m-MTDATA/T2T bilayer at various temperatures: **a** 100 K, **b** 150 K, **c** 200 K, **d** 250 K, and **e** 290 K. Black and red markers correspond to $XF_{m\text{-}MT}$ and $XP_{m\text{-}MT/T2T}$, respectively.

Supplementary Note 11: FWHM of PL spectra of m-MTDATA/T2T bilayer as a function of P_{in}

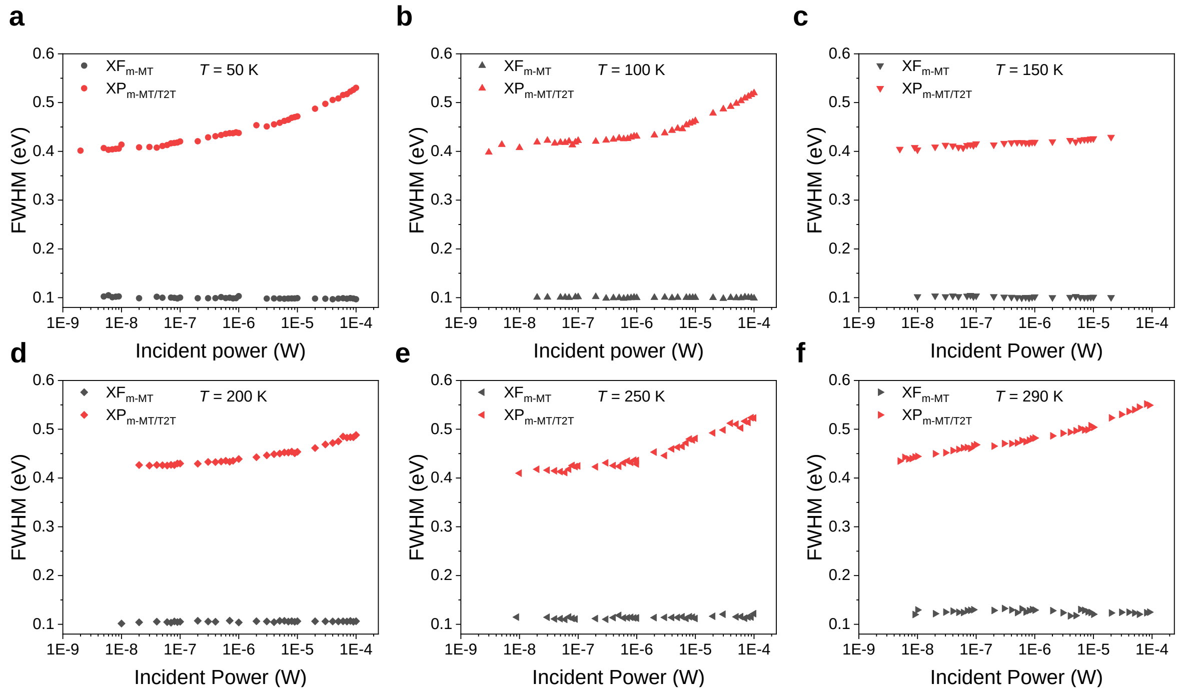

Supplementary Fig. 13 | FWHM of PL spectra of m-MTDATA/T2T bilayer. Incident excitation power dependence of FWHM of $XF_{m\text{-}MT}$ and $XP_{m\text{-}MT/T2T}$ of m-MTDATA/T2T bilayer at various low temperatures: **a** 50 K, **b** 100 K, **c** 150 K, **d** 200 K, **e** 250 K, and **f** 290 K. Black and red markers correspond to $XF_{m\text{-}MT}$ and $XP_{m\text{-}MT/T2T}$ of the m-MTDATA/T2T bilayer, respectively.

Supplementary Note 12: Time-resolved PL decay curves and average lifetime (τ_{avg}) of T2T layer at various temperatures

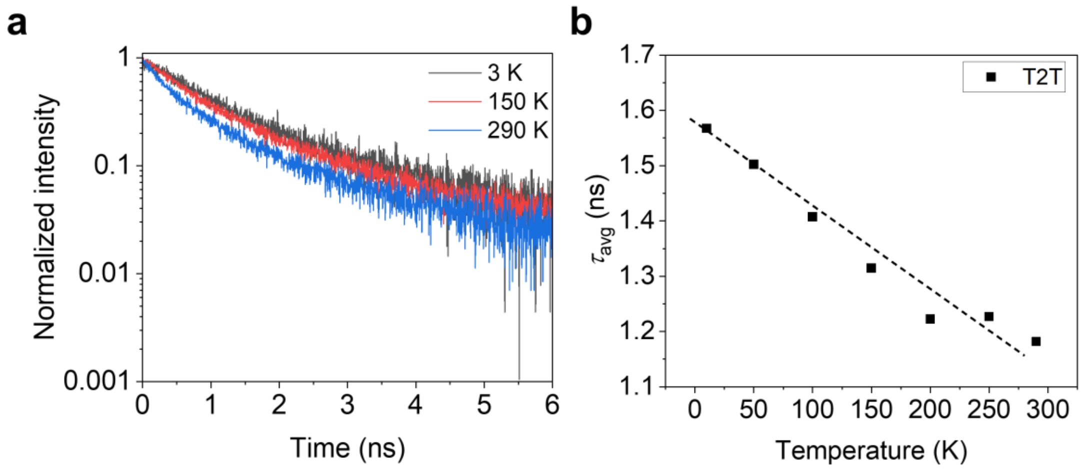

Supplementary Fig. 14 | Time-resolved PL decay of T2T. a Time-resolved PL decay curves of T2T layer at 3 K (black curve), 150 K (red curve), and 290 K (blue curve). **b** Averaged exciton lifetime (τ_{avg}) of T2T at various low temperatures.

Time-resolved PL (tr-PL) decay curves were analyzed by fitting them to the bi-exponential decay function, $y(t) = a_1 \exp[-t/\tau_1] + a_2 \exp[-t/\tau_2]$.

The averaged lifetimes were calculated using the intensity-weighted average lifetime, $\tau_{avg} = \Sigma(a_i\tau_i^2)/\Sigma(a_i\tau_i)$ $(i = 1, 2)$. The fitting results are presented in Supplementary Table 2.

Supplementary Table 2 | Fitting results of tr-PL decay curves for the prompt and delayed components of XP at various temperatures.

**Prompt component of XP**							
**temperature (K)**	**3**	**50**	**100**	**150**	**200**	**250**	**290**
a_1	0.59	0.50	0.54	0.52	0.47	0.47	0.34
τ_1 (ns)	1.49	1.55	1.26	1.31	1.19	1.06	0.93
a_2	0.41	0.50	0.46	0.48	0.53	0.53	0.66
τ_2 (ns)	7.54	8.41	6.36	5.95	4.85	4.23	3.74
τ_{avg} (ns)	6.20	7.33	5.39	5.06	4.19	3.66	3.43
**Delayed component of XP**							
**temperature (K)**	**3**	**50**	**100**	**150**	**200**	**250**	**290**
a_1	0.37	0.55	0.57	0.55	0.58	0.63	0.66
τ_1 (μs)	1.34	1.56	1.21	1.12	1.15	1.05	0.82
a_2	0.63	0.45	0.43	0.45	0.42	0.37	0.34
τ_2 (ns)	5.15	5.40	4.81	4.49	4.54	3.94	2.87
τ_{avg} (μs)	4.65	4.41	3.90	3.69	3.67	3.04	2.14

Supplementary Note 13: Schematic illustration of electrical-driven $XP_{m\text{-}MT/T2T}$

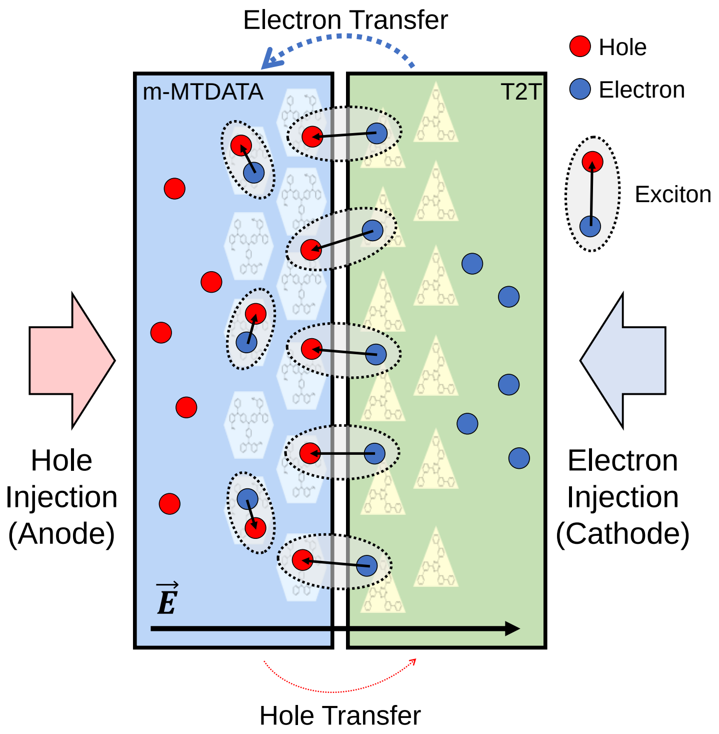

Supplementary Fig. 15 | Schematic illustration of electrical-driven XPs in m-MTDATA/T2T bilayer OLED. The black arrow from electron to hole indicates the direction of exciton dipole moments.

Supplementary Note 14: EL spectra of m-MTDATA:T2T CDL OLED

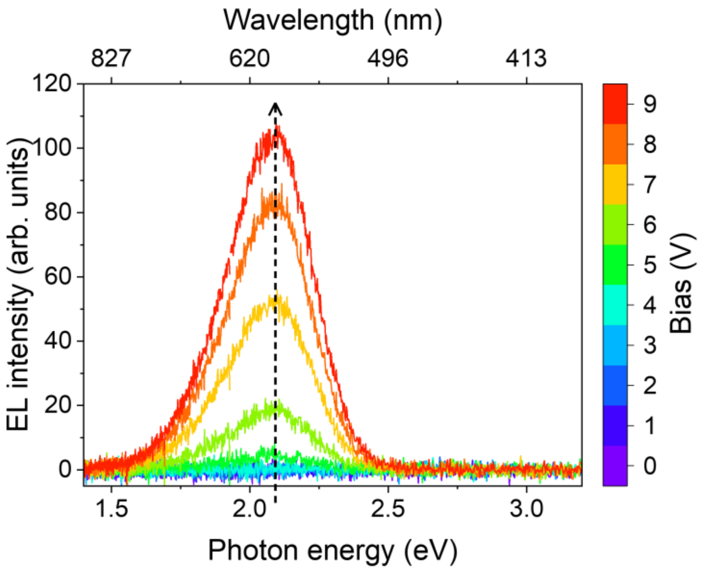

Supplementary Fig. 16 | EL spectra of m-MTDATA:T2T CDL OLED in various applied biases.

Supplementary Note 15: PL peak positions of exciplexes in the bilayer and CDL

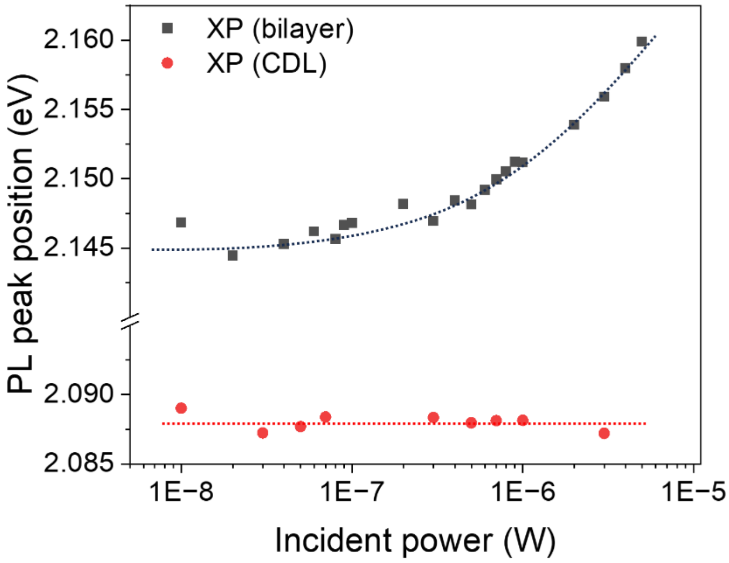

Supplementary Fig. 17 | PL peak positions of exciplexes. Incident power dependence of PL peak positions of XPs from m-MTDATA/T2T BL (black) and m-MTDATA:T2T CDL (red) at 3 K. The dotted lines are eye-guided lines.

Supplementary Note 16: Device characteristics of BL and CDL OLEDs

We measured device characteristics such as current density–voltage–luminance (*I*–*V*–*L*), external quantum efficiency (EQE) of OLEDs using BL and CDL of donor m-MTDATA and acceptor T2T. Supplementary Fig. 18a shows *I*–*V*–*L* characteristic curves of the BL (red curves) and CDL (black curves) OLEDs. The BL OLED showed more stable *I*–*V*–*L* properties, whereas the CDL OLED showed decreased luminance and current density with an applied bias over 6.5 V and 9.5 V, respectively. The interlayer junction of the BL and the randomized intermolecular junction of the CDL in the OLEDs induced a difference in device stability, probably because of the relatively homogeneous local electric field near the junction in the BL compared with the inhomogeneous local electric field of the random distribution of the donor m-MTDATA and acceptor T2T molecules in the CDL. The large current of the CDL OLED originates from the easy injection and formation of a hole channel from the ITO to the active layer.

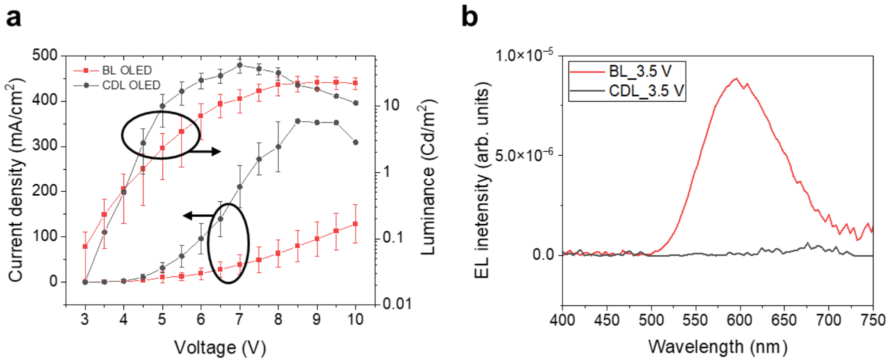

Supplementary Fig. 18 | Characteristics of OLEDs. a *I*–*V*–*L* curves of BL (red) and CDL (black) OLEDs with error bars. **b** EL spectra of BL (red) and CDL (black) OLEDs in a bias of 3.5 V. The error bars mean standard deviation.

Notably, the EL spectrum of the BL OLED corresponding to the XPs was observed at λ_{em} = 585 nm at a relatively low bias (=3.5 V), whereas that of the CDL OLED was very weak,

as shown in Supplementary Fig. 18b. This suggests the easy formation of CT excitons with a low bias at the BL interface of the donor m-MTDATA and acceptor T2T.

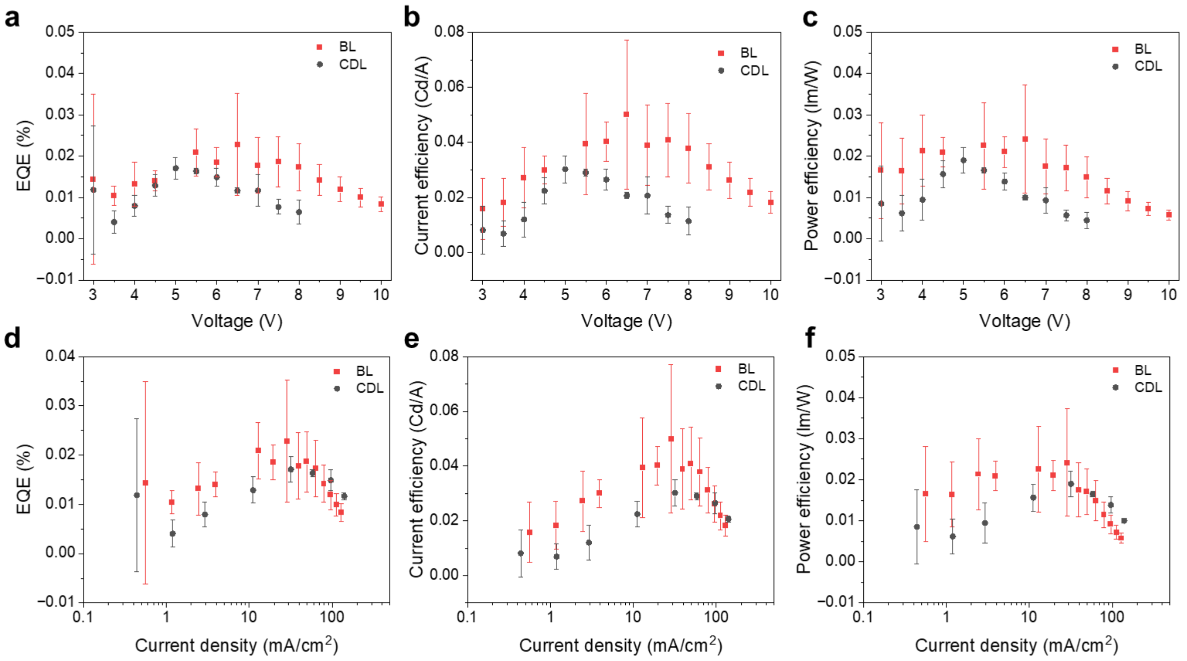

Supplementary Fig. 19 | Efficiencies of OLEDs. a and d EQE, **b and e** CE, **c and f** PE of BL (red) and CDL (black) OLEDs as functions of bias and current density with error bars. The error bars mean standard deviation.

Supplementary Fig. 19 shows the EQE, current efficiency (CE), and power efficiency (PE) as functions of bias and current density for the BL (red) and CDL (black) OLEDs. It is noted that the OLEDs for Supplementary Fig. 19 are new batch devices. The overall efficiency of the BL OLED was slightly higher than that of the CDL OLED in applied voltage dependence (Supplementary Fig. 19a–c). The EQE, CE, and PE of BL OLEDs showed comparable efficiencies with those of CDL OLEDs at high current density levels considering error bars (over 10 mA/cm^2, Supplementary Fig. 19d-f). In low current regime, the injected electron-hole recombination rate for radiative decay in the BL OLED is higher than that of the blended D-A

molecules in the CDL OLED due to well defined D/A interface. The low EQE, CE, and PE of the BL and CDL OLEDs can be attributed to low outcoupling by the wide-angle emission of XP, power loss by hole leakage, and/or low charge balancing caused by the lack of functional layers such as the electron injection layer (EIL). A clear correlation between the EL peak positions with increasing bias and the dipole orientation of excitons/exciplexes has been discussed with Fig. 6c. The efficiencies of BL and CDL OLEDs (Supplementary Fig. 19d-f) at high current density levels did not show considerably differences (within error bars) related to the dipole orientation of exciton species.

The photoluminescence quantum yield (PL QY) was measured using an integrating sphere (Newport, 819C-IS-5.3) and a 325-nm-fiber-coupled LED. Supplementary Fig. 20 shows the PL spectra of LED, m-MTDATA/T2T BL (50 nm/50 nm), and m-MTDATA:T2T CDL (1:1, 50 nm). The calculated PL QY values for each sample were 26.4% for XP (with excimers (XM)) for BL and 46.9% for CDL (XP only). These PL QY values for the XPs were similar to those obtained using m-MTDATA and/or T2T[6,7]. The higher PL QY of the CDL than that of the BL is due to the higher density of CT excitons in the bulky CDL compared to those from the interface of the BL.

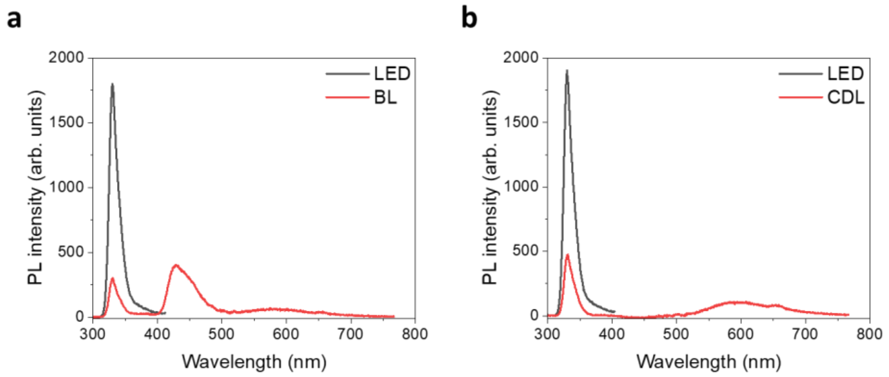

Supplementary Fig. 20 | PL spectra of 325-nm-fiber-coupled LED (black curves), **a** m-MTDATA/T2T BL (red curve), and **b** m-MTDATA:T2T CDL (red curve).

Supplementary Note 17: Comparison of LCM PL spectra of m-MTDATA layer and m-MTDATA/T2T bilayer

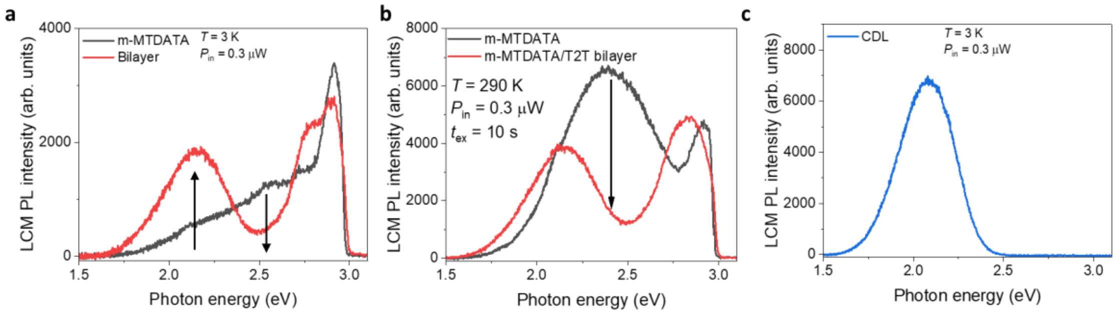

Supplementary Fig. 21 | Comparison of LCM PL spectra of m-MTDATA layer and m-MTDATA/T2T bilayer. LCM PL spectra of m-MTDATA layer (black curves) and m-MTDATA/T2T bilayer (red curves) **a** 3 K and **b** 290 K. **c** LCM PL spectrum of m-MTDATA:T2T CDL at 3 K.

The efficient ET between $XP_{m\text{-}MT/T2T}$ (~ 2.15 eV at 3 K) and $XM_{m\text{-}MT}$ (~ 2.5 eV at 3 K) owing to the alignment dipole moments (κ^2 factor) results in the increase and decrease in PL intensity of the XP and XM, respectively As shown in Supplementary Fig. 21a and b, PL peak of XPs in m-MTDATA/T2T bilayer was considerably enhanced, decreasing that of XM, while PL peak of XF was weakly changed. As shown in Supplementary Fig. 3a, there was almost no characteristic absorption peak in the range of 450–550 nm after the hybridization of m-MTDATA and T2T. These results indicate that both FRET and CT-related DET effects between parallelly aligned XPs and XMs are involved in the MTDATA/T2T BL. In contrast, XPs were dominant in the PL spectrum of the m-MTDATA:T2T CDL with a very weak XF PL peak, as shown in Supplementary Fig. 21c. This suggests that the DET effect was relatively dominant for the D-A CDL because of the much larger overlap of wave functions from the random and

opposite dipole moments of XPs from the blending donor and acceptor molecules. In terms of OLED performance, the EL peaks of XP for the BL OLED were rapidly enhanced and blue-shifted with increasing applied bias. In contrast, those of the CDL OLED were very weak and not shifted, as shown in Fig. 6 and Supplementary Fig. 16. This originates from the different DET efficiencies and configurations of the dipole orientations of XPs, XMs, and XPs in our organic m-MTDATA and T2T BL and CDL systems.

Supplementary Note 18: Incident excitation power dependence of PL intensity

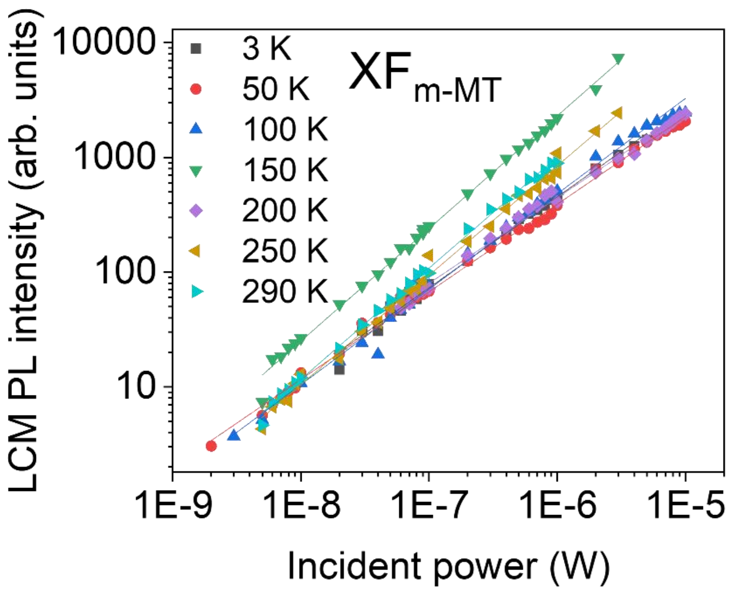

Supplementary Fig. 22 | Incident power dependence of PL intensity. Incident excitation power dependence of PL intensity (spectral area) of XF_{m-MT} measured from m-MTDATA/T2T bilayer at various low temperatures. A linear fitting by applying $I_{PL} = I_0 P_{in}^{\alpha}$ revealed that the values of exponent α of the PL intensity for the XF_{m-MT} increased from 0.79 (3 K) to 0.97 (290 K).

Supplementary References

1. Krustok, J. et al. K. The role of spatial potential fluctuations in the shape of the PL bands of multinary semiconductor compounds. *Phys. Scr.* **T79**, 179–182 (1999).

2. Yakushev, M. V. et al. Effects of Ar+ etching of $Cu_2ZnSnSe_4$ thin films: An X-ray photoelectron spectroscopy and photoluminescence study. *J. Vac. Sci. Technol. B* **36**, 061208 (2018).

3. Schuller, J. A., et al. Orientation of luminescent excitons in layered nanomaterials. *Nat. Nanotechnol*. **8**, 271-276 (2013)

4. Salehi, A., et al. Recent Advances in OLED Optical Design. *Adv. Func. Mater*. **29**, 1808803 (2019).

5. Gujral, A. et al. Vapor-deposited glasses with long-range columnar liquid crystalline order. *Chem. Mater*. **29**, 9110–9119 (2017).

6. Cocchi, M. et al. Efficient exciplex emitting organic electroluminescent devices. *Appl. Phys. Lett*. **80**, 2401-2403 (2002)

7. Zhang, M. et al. Tricomponent Exciplex Emitter Realizing over 20% External Quantum Efficiency in Organic Light-Emitting Diode with Multiple Reverse Intersystem Crossing Channels. *Adv. Sci.* **6**, 1801938 (2019)
